# The role of advanced MRI in the development of treat-to-target therapeutic strategies, patient stratification and phenotyping in rheumatoid arthritis

**DOI:** 10.1186/s41927-020-00131-w

**Published:** 2020-05-28

**Authors:** Ali Mobasheri, Mark Hinton, Faiq Shaikh, Olga Kubassova

**Affiliations:** 1 Research Unit of Medical Imaging, Physics and Technology, Faculty of Medicine, University of Oulu, PO Box 5000, Aapistie 5 A, FIN-90230 Oulu, Finland; 2 Department of Regenerative Medicine, State Research Institute Centre for Innovative Medicine, Santariskiu 5, LT-08406 Vilnius, Lithuania; 3 Department of Orthopedics and Department of Rheumatology and Clinical Immunology, University Medical Center Utrecht, 508 GA, Utrecht, The Netherlands; 4 Centre for Sport, Exercise and Osteoarthritis Research Versus Arthritis, Queen’s Medical Centre, Nottingham, NG7 2UH UK; 5 IAG, Image Analysis Group, London, UK

**Keywords:** Rheumatoid arthritis, Dynamic contrast enhanced (DCE), Magnetic resonance imaging (MRI), Bone marrow edema (BME), Synovitis

## Abstract

In this commentary, we discuss the potential of advanced imaging, particularly Dynamic Contrast Enhanced (DCE) magnetic resonance imaging (MRI) for the objective assessment of the inflammatory process in rheumatoid arthritis (RA). We emphasise the potential of DCE-MRI in advancing the field and exploring new areas of research and development in RA. We hypothesize that different grades of bone marrow edema (BME) and synovitis in RA can be examined and monitored in a more sensitive manner with DCE-MRI. Future treatments for RA may benefit from the application of enhanced imaging of BMEs and synovitis. DCE-MRI may also facilitate enhanced stratification and phenotyping of patients enrolled in clinical trials.

Rheumatoid Arthritis (RA) is an inflammatory disease affecting about 1.3 million adults in the United States and many millions more across the world. RA causes pain, swelling, stiffness, and loss of function in synovial joints and has a huge socioeconomic impact due to the high morbidity associated with it. It is estimated that between 0.5 and 1% of the human population is affected worldwide, and between 25 and 50 new RA cases evolve in a population of 100,000 [1]. RA can manifest itself rapidly and often in a symmetrical pattern, typically affecting. The wrist joints and the finger joints closest to the handRA occurs in all races and ethnic groups [2]. The cause of RA is not known, but it is believed to be an autoimmune disease, and has predilection for the female gender [3].

New patients with RA require a rapid and definitive diagnosis and treatment initiation with disease-modifying antirheumatic drugs (DMARDs) to retard or stop progression, stimulate remission, control disease manifestations and reduce the overall disease burden [4]. Advanced imaging approaches, particularly magnetic resonance imaging (MRI) has an important role to play in the clinical management and in the evaluation of new drugs in clinical trials. In a recent study by Møller-Bisgaard, et al., it was reported on whether the integration of MRI into clinical rheumatology practice can enhance strategies for monitoring disease activity and determining whether the therapy being tested is effective in slowing down joint damage and disease progression [5]. The key objective of this study (IMAGINE-RA randomized trial) was to determine whether an MRI-guided treat-to-target strategy versus a conventional clinical treat-to-target strategy improves outcomes in patients with RA in clinical remission. The presence of bone marrow edema (BME) in the wrist or MCP joints was used as a marker to escalate a predefined treatment algorithm versus a conventional clinical treat-to-target strategy can improve outcomes in patients with RA in clinical remission. BME occurs in various forms of inflammatory and non-inflammatory arthritis and probably represents infiltration of inflammatory cells and vascular perfusion changes within the bone marrow. BME is common in early RA and is associated with erosive progression and poor functional outcomes [6]. It is also well established that the location and extent of BME in psoriatic arthritis (PsA) is different from those seen in RA and osteoarthritis (OA) [7, 8], suggesting that treatment monitoring strategies can benefit from MRI-based biomarkers of BME. However, the recent study by Møller-Bisgaard, et al.gues showed that MRI-guided treat-to-target strategy using BME compared with a conventional treat-to-target strategy did not result in improved disease activity remission rates and it did not reduce radiographic progression over 2 years in this particular patient cohort. Although this negative study does not support the use of BME as a biomarker in MRI-guided strategy for treating patients with RA in clinical remission and low disease activity, it does offer exciting opportunities for future research.

Despite the study’s conclusion that making treatment decisions based upon BME on MRI, is not clinically helpful, there are alternative and more promising possibilities.

Given that BME may serve as a biomarker of inflammation in RA and other inflammatory arthritides, this phenomenon can be better understood with more advanced imaging sequences such as Dynamic Contrast Enhanced (DCE)-MRI [9]. This MR sequence of rapidly acquired images after the Gadolinium-based contrast agent injection was collected as a part of the IMAGINE-RA imaging protocol. Due to its dynamic nature (as opposed to static acquisition), DCE-MRI visualises the vascularisation levels of the oedema lesions, seving as a biomarker of perfusion and inflammation. Brown, et al. suggested that the best predictor of progressive erosion in RA is Doppler ultrasound [10]. However, no multivariate analysis was performed in that study. As previously shown by Hodgson et al. [11, 12], DCE-MRI can be used to quantify the perfusion and treatment changes in the bone compartment. This approach can potentially help to distinguish between inflammation, repair or trauma in the bone, which all look oedema-like on static MRI sequences and reveal treatment responses as measured with the AI-driven methodologies that are based on the objective quantitative assessment of DCE-MRI time vs intensity curves [9]. As these subsequent studies that have shown MR-based assessment of inflammation to be a strong, independent predictor of disease progression, the role of BME as an DCE-MRI biomarker should be investigated.

There is an opportunity here to use DCE-MRI image datasets from the same or similar trials to explore the heterogeneity of BME and synovial perfusion/inflammation in order to better phenotype RA patients and show vascular responses following various treatment regimens that can complement the widespread and validated static scoring systems such as RAMRIS. Furthermore, there may be a unique opportunity to examine the overlap between RA and cardiovascular diseases that impact on vascular perfusion, especially in bone, implicating endothelial dysfunction and angiogenesis impairment in the cardiovascular system [13] and the ageing vasculature of arthritic joints [14].

In summary, we suggest that advanced imaging can be a potentially important role to facilitate disease diagnosis, and possibly monitor disease progression, which may then enhance clinical trials of new RA treatments. Bone marrow may indeed be an important site for looking at these pathological changes that drive joint damage and destruction in RA and other joint diseases such as PsA and even in the inflammatory phenotypes of OA [15, 16]. It has been proposed that imaging remission should only be selected as a target if it can be convincingly demonstrated that it can be treated and that the clinical outcome for patients will be improved by trying to achieve imaging remission in addition to clinical remission [17]. Our hypothesis is that different grades of BME and synovitis examined with DCE-MRI may help predict erosive progression regardless of treatment strategy in patients with RA in clinical remission and low disease activity. For these purposes, DCE-MRI may prove to be a useful tool to test, in a more targeted way, precision treatments in RA. Using such advanced imaging approaches can help identidy key phenotypic biomarkers of disease, which can potentially lead to better outcomes in smarter clinical trials and in the rheumatology clinics of the future [18].

## Data Availability

No patient data was used.
